# Effects of pre-pregnancy body mass index on pregnancy and perinatal outcomes in women based on a retrospective cohort

**DOI:** 10.1038/s41598-021-98892-y

**Published:** 2021-10-06

**Authors:** Donghua Xie, Wenzhen Yang, Aihua Wang, Lili Xiong, Fanjuan Kong, Zhiyu Liu, Zhiqun Xie, Hua Wang

**Affiliations:** 1 Department of Information Management, Maternal, Child Health Hospital of Hunan Province, 58 Xiangchun Road, Changsha, 410078 Hunan China; 2 Department of Health Management, Maternal, Child Health Hospital of Hunan Province, 58 Xiangchun Road, Changsha, 410078 Hunan China; 3 NHC Key Laboratory of Birth Defect for Research and Prevention, Hunan Provincial Maternal and Child Health Care Hospital, 58 Xiangchun Road, Changsha, 410078 Hunan China

**Keywords:** Health care, Public health, Weight management

## Abstract

To investigate the prevalence of underweight, overweight and obesity as defined by pre-pregnancy body mass index (BMI) and the relationship between pre-pregnancy BMI and pregnancy and perinatal outcomes in women based on a retrospective cohort. Women registered via the Free Pre-pregnancy Health Check (FPHC) program from 2017 to 2019 in Hunan Province, China, were included to the study cohort. The data regarding maternal characteristics, pregnancy outcomes, and infant characteristics were retrieved from the surveillance system of the FPHC program. Logistic regressions were performed to calculate odds ratios (ORs) and adjusted odds ratios (AORs) with 95% confidence intervals (95% CIs) to assess the associations between pre-pregnancy BMIs and the outcomes. Among a total of 398,368 women, 54,238 (13.62%) were underweight (BMI < 18.5 kg/m^2^), 51,251 (12.87%) were overweight (24.0 kg/m^2^ ≤ BMI < 28.0 kg/m^2^), and 10,399 (2.61%) were obese (BMI ≥ 28.0 kg/m^2^). Underweight occurred more commonly in the 20–24 years old (17.98%), Han Chinese (13.89), college-educated (16.09%), rural (13.74%), and teacher/public servant/office clerk (16.09%) groups. Obesity occurred more often in the older than 35-year-old (4.48%), minority (3.64%), primary school or below (4.98%), urban (3.06%), and housewife (3.14%) groups (*P* < 0.001). Compared with the normal BMI group, underweight was associated with increased risk of low birth weight (LBW) (AOR = 1.25) and small-for-gestational age (SGA) (AOR = 1.11), but protected against gestational hypertensive disorder (GHD) (AOR = 0.85), gestational diabetes mellitus (GDM) (AOR = 0.69), macrosomia (AOR = 0.67), post-term pregnancy (AOR = 0.76), and cesarean-section (AOR = 0.81). Overweight and obesity were associated with increased risk of GHD (AOR = 1.28, 2.47), GDM (AOR = 1.63, 3.02), preterm birth (AOR = 1.18, 1.47), macrosomia (AOR = 1.51, 2.11), large-for-gestational age (LGA) (AOR = 1.19, 1.35), post-term pregnancy (AOR = 1.39, 1.66), and cesarean- section (AOR = 1.60, 2.05). Pre-pregnancy underweight is relatively common in Hunan Province, China. Pre-pregnancy underweight to some extent is associated with better maternal outcomes, but it has certain adverse effects on neonatal outcomes. Pre-pregnancy overweight, especially obesity, has a substantial adverse effect on pregnancy and perinatal outcomes.

## Introduction

Overweight and obesity has reached epidemic proportions, with over 4 million people dying each year as a result of being overweight or obese according to the global burden of disease in 2017^1^. Therefore, many studies have paid close attention to the adverse outcomes associated with overweight and obesity^2–4^. Studies have shown that a higher pre-pregnancy body mass index (BMI) is associated with an increased risk of gestational diabetes, cesarean-section, postpartum hemorrhage, fetal macrosomia, and other maternal and neonatal outcomes^5,6^. By contrast, the problem of underweight has been underestimated. Currently, many young women have a strong desire to keep slim. In China, 3.0% (95% CI 2.6–3.4%) women being underweight consider themselves overweight^7^. The studies by N.J. Sebire and colleagues showed that low maternal BMI was associated with an increased prevalence of certain pregnancy complications, with preterm delivery and low birth weight (LBW) being most notable^8^. In addition, the sample sizes of previous studies did not allow for further assessment of the obesity subgroup, and the impact of pre-pregnancy BMI on adverse outcomes may not have been estimated accurately^9^. Moreover, the impact of pre-pregnancy BMI on adverse pregnancy outcomes varies in different countries or geographic areas because of the differences in the characteristics of populations, culture, medical condition and body fat percentage^10^.

China is a developing country. Hunan Province is in the central south of China with a large population of over 70 million. There are approximately 700,000 newborn infants every year in Hunan Province. The local young women are eager for a slim body type, and are fond of spicy foods. Additionally, there are a few rural areas in Hunan Province that may be associated with underweight. Therefore, the distribution of pre-pregnancy BMI in Hunan Province may be different from the average level in China and the world.

In 2010, the Health Ministry of China (HMC) initiated a Free Pre-pregnancy Health Check (FPHC) program to all couples planning for conception. For this program, HMC set up a surveillance system to collect all the data including the results of pre-pregnancy health examinations, follow-up of early pregnancy, follow-up of pregnancy outcome. By using this system, we investigated the prevalence of pre-pregnancy underweight, normal weight, overweight and obesity as defined by BMI in a large population of women in Hunan Province, and the relationship between pre-pregnancy BMI and pregnancy and perinatal outcomes.

## Methods

### Study design and population

We performed a retrospective cohort study of women whose information was recorded in the FPHC surveillance system from 2017 to 2019 in Hunan Province, China. With respect to the FPHC program, the maternal and child health care hospitals at all levels were responsible for conducting the examinations and follow-ups. The delivery hospitals were responsible for reporting the birth defect cards online and providing delivery information to local maternal and child health care hospitals. All FPHC archives were subjected to the national external quality assessment twice a year. The HMC required the maternal and child health care hospitals to create paper files for every couple that received FPHC and input these files into the FPHC surveillance system, which uses the personal unique identification number for tracking. Therefore, the system can provide the pre-pregnancy to post-delivery information for all registered women. The information in the system was audited by the provincial maternal and child health care hospital with authorization from the local health administrative department.

During the period of 2017–2019, 431,412 couples received FPHC and had given birth, accounting for 20.27% of the total maternal women of Hunan Province. The exclusion criteria were as follows: twin or multiple pregnancy, report of spontaneous conception or stillbirth, pre-existing diabetes mellitus or hypertension, thyroid disorders, virus infection, tuberculosis, history of cesarean-section and missing BMI. Finally, 398,368 women were included in this study.

This study was approved by the Medical Ethics Committee of Hunan Provincial Maternal and Child Health Care Hospital in accordance with the relevant guidelines and regulations. All women who received FPHC signed the letter of informed consent. All personal information was de-identified before the data being analyzed.

### Data collection

All data used in this study were previously deposited in the FPHC surveillance system. We logged into the FPHC system webpage to search the maternal and infant data, and finally extracted and summarized the data in an Excel table. The following variables were included: maternal characteristics (weight, height, BMI, ethnic groups, education, age, area of residence, occupation, smoking behavior, alcohol consumption behavior, history of spontaneous conception or stillbirth, diabetes mellitus or hypertension, thyroid disorders, viral infection, tuberculosis, history of cesarean section), infant characteristics (sex, birth weight, gestational weeks at birth, outcome, small-for-gestational age (SGA), large-for-gestational age (LGA), birth defects (BDs)), and pregnancy outcomes (cesarean-section, preterm birth, postterm pregnancy, abortion, still birth, LBW, macrosomia, hypertensive disorder (GHD), gestational diabetes mellitus (GDM)).

### Definition of variables

BMI (kg/m^2^) was calculated as the ratio of the weight (kg) divided by height squared (m^2^) in terms of the data for women from the FPHC program. Based on pre-pregnancy BMI, women were divided into four groups as follows: underweight (BMI < 18.5 kg/m^2^), normal weight (18.5 kg/m^2^ ≤ BMI < 24.0 kg/m^2^),overweight (24.0 kg/m^2^ ≤ BMI < 28.0 kg/m^2^), and obese (BMI ≥ 28.0 kg/m^2^). This classification followed the criteria suggested by the Working Group on Obesity in China, which were thought to more sensitive and specific for Chinese women^6^. For statistical comparisons, women with normal BMI (18.5–24.0 kg/m^2^) were used as the reference group.

LBW was defined as birth weight < 2500 g, whereas fetal macrosomia was defined as birth weight > 4000 g regardless of gestational age. Preterm delivery was defined as birth at < 37 completed weeks of gestation, whereas postterm delivery was defined as birth at ≥ 42 completed weeks of gestation. GDM diagnosis was based on the published criteria (2014) by the National Health and Family Planning Commission of the People’s Republic of China. When 75-g OGTT outcomes showed that the 0-h plasma glucose value (PGV) ≥ 5.1 mmol/L or 1-hPGV ≥ 10.0 mmol/L or 2-hPGV ≥ 8.5 mmol/L, the pregnant women were diagnosed with GDM. 75 g OGTT was performed between the 24th and 28th weeks of gestation for all pregnant women who were not previously known to suffer from diabetes^3^. The test results were still valid even after 28 weeks. GHD was defined as systolic blood pressure ≥ 140 mmHg and/or diastolic blood pressure ≥ 90 mmHg at least two occasions and four or more hours apart arising after 20 weeks of gestation^11^. The diagnosis of BDs was based on the Chinese National Criteria of Birth Defects, including structural and functional malformations. SGA was defined as birth weight below the 10th percentile of mean weight corrected for fetal sex and gestational age, and LGA was defined as birth weight above the 90^th^percentile of mean weight corrected for fetal sex and gestational age.

### Statistical analysis

The data were analyzed using SPSS 21.0 (SPSS, Chicago, IL). Continuous variables were expressed as mean ± standard deviation (SD), and compared using one-way ANOVA test. Categorical variables were compared by Pearson’s chi-square test. Logistic regression was conducted to calculate odds ratios (ORs) and adjusted odds ratios (AORs) with 95% confidence intervals (95% CIs) to assess the associations between pre-pregnancy BMIs and the outcomes. The adjusted variables included maternal age, ethnic group, education, area of residence, occupation, smoking, passive smoking, alcohol drinking.

## Results

### Maternal characteristics and the prevalence of the pre-pregnancy BMI groups

As defined by pre-pregnancy BMI, 54,238 (13.62%) women were underweight, 282,480(70.91%) women were normal-weight, 51,251 (12.87%) women were overweight, and 10,399 (2.61%) women were obese. Maternal age ranged from 16 to 52 years old, and 92.26% of the women were between 20 and 34 years old. The majorities (88.79%) of the women were Chinese of Han descent, and 68.81% of the women had a college or higher education. The registered residences were mainly (93.20%) rural and 61.79% women were farmers. A few women had serious smoking (0.1%) and alcohol consumption (0.67%) behaviors, and 3.4% of the women were passive smokers. Obesity occurred more often in individuals older than 35 years (4.48%) versus those aged ≤ 35 years (2.15% to 3.25%), minorities (3.64%) versus Han Chinese (2.48%), individuals with primary school or lower education (4.98%) versus higher educations (1.52% to 3.19%), urban areas (3.06%) versus rural areas (2.58%), and housewives (3.14%) versus occupational women (1.63% to 2.64%) (Pearson chi-square test, all *P* < 0.001). Underweight occurred more often in individuals aged 20–24 years (17.98%) versus those in other age groups (5.09% to 14.37%), Han Chinese (13.89%) versus minorities (11.47%), individuals with college education (16.09%) versus lower education levels (12.25% to 15.16%), rural areas (13.74%) versus urban areas (11.96%), and teachers/public servants/office clerks (16.09%) versus farmer, worker, merchant, housewife and service personnel (13.66% to 15.19%) (Pearson chi square test, all *P* < 0.001) (Table 1).

**Table 1:** Maternal characteristics and the prevalence of the pre-pregnancy BMI groups.

Characteristics	BMI < 18.5 kg/m^2^ (n = 54,238)			18.5 kg/m^2^ ≤ BMI < 24.0 kg/m^2^(n = 282,480)		24.0 kg/m^2^ ≤ BMI < 28.0 kg/m^2^ (n = 51,251)			BMI ≥ 28.0 kg/m^2^ (n = 10,399)			Total	%	X^2^	*P*
	n	%	Prevalence (%)	n	%	n	%	Prevalence (%)	n	%	Prevalence (%)	n			
**Maternal age**															
< 20	16	0.03	12.6	92	0.03	16	0.03	12.6	3	0.03	2.36	127	0.03		
20 ~	18,664	34.41	17.98	72,498	25.66	10,410	20.31	10.03	2234	21.48	2.15	103,806	26.06		
25 ~	26,544	48.94	14.37	132,100	46.76	21,807	42.55	11.81	4225	40.63	2.29	184,676	46.36	7926.581	< 0.001
30 ~	7434	13.71	9.4	56,532	20.01	12,520	24.43	15.84	2572	24.73	3.25	79,058	19.85		
35 ~	1537	2.83	5.09	20,896	7.4	6437	12.56	21.3	1353	13.01	4.48	30,223	7.59		
Missing	43			362	0.13	61	0.12		12	0.12		478	0.12		
**Ethnic groups**															
Han Chinese	49,115	90.55	13.89	251,571	89.06	44,242	86.32	12.51	8773	84.36	2.48	353,701	88.79	707.768	< 0.001
Minority	5123	9.45	11.47	30,909	10.94	7009	13.68	15.69	1626	15.64	3.64	44,667	11.21		
**Education**															
Primary School or Below	619	1.14	10.07	4233	1.5	986	1.92	16.05	306	2.94	4.98	6144	1.54		
Junior high school	14,472	26.68	12.25	82,662	29.26	17,218	33.6	14.58	3770	36.25	3.19	118,122	29.65		
High School or Technical School	21,611	39.84	14.29	108,116	38.27	18,066	35.25	11.94	3470	33.37	2.29	151,263	37.97	3510.917	< 0.001
College	12,854	23.7	16.09	57,187	20.24	8454	16.5	10.58	1395	13.41	1.75	79,890	20.05		
Bachelor and above	1994	3.68	15.16	9720	3.44	1243	2.43	9.45	200	1.92	1.52	13,157	3.3		
Missing	2688	4.96		20,562	7.28	5284	10.31		1258	12.1		29,792	7.48		
**Area of residence**															
Urban	3238	5.97	11.96	18,925	6.7	4084	7.97	15.08	828	7.96	3.06	27,075	6.8	196.147	< 0.001
Rural	51,000	94.03	13.74	263,555	93.3	47,167	92.03	12.7	9571	92.04	2.58	371,293	93.2		
**Occupation**															
Farmer	33,633	62.01	13.66	175,194	62.02	31,026	60.54	12.6	6304	60.62	2.56	246,157	61.79		
Worker	2076	3.83	14.36	10,112	3.58	1887	3.68	13.05	382	3.67	2.64	14,457	3.63		
Merchant	1958	3.61	15.19	9055	3.21	1571	3.07	12.19	302	2.9	2.34	12,886	3.23		
Teacher/public servant/office clerk	5010	9.24	16.09	22,569	7.99	3044	5.94	9.78	507	4.88	1.63	31,130	7.81	2842.649	< 0.001
Housewife	2161	3.98	13.86	10,826	3.83	2116	4.13	13.57	490	4.71	3.14	15,593	3.91		
Service personal	3769	6.95	13.96	19,735	6.99	2986	5.83	11.06	505	4.86	1.87	26,995	6.78		
Others	2067	3.81	17.92	7840	2.78	1367	2.67	11.85	263	2.53	2.28	11,537	2.9		
Missing	3564	6.57		27,149	9.61	7254	14.15		1646	15.83		39,613	9.94		
**Smoking**															
Yes	57	0.11	14.11	260	0.09	67	0.13	16.58	20	0.19	4.95	404	0.1		
No	53,929	99.43	13.63	280,481	99.29	51,019	99.55	12.89	10,341	99.44	2.61	395,770	99.35	99.767	< 0.001
Missing	252	0.46		1739	0.62	165	0.32		38	0.37		2194	0.55		
**Passive smoking**															
Yes	2183	4.02	16.12	9194	3.25	1834	3.58	13.55	327	3.14	2.42	13,538	3.4	88.192	< 0.001
No	51,813	95.53	13.54	271,588	96.14	49,243	96.08	12.87	10,033	96.48	2.62	382,677	96.06		
Missing	242	0.45		1698	0.6	174	0.34		39	0.38		2153	0.54		
**Alcohol consumption**															
Yes	383	0.71	14.42	1824	0.65	378	0.74	14.23	71	0.68	2.67	2656	0.67		
No	53,594	98.81	13.62	278,907	98.74	50,690	98.91	12.88	10,292	98.97	2.62	393,483	98.77	8.15	0.078
Missing	261	0.48		1749	0.62	183	0.36		36	0.35		2229	0.56		

### Infant characteristics in the distinct groups of pregnant women stratified by pre-pregnancy BMI

Overall, 26.98% infants were born through cesarean-section, 52.52% infants were male, 99.54% infants survived, 0.4% infants were stillborn, and 0.06% infants died within 42 days. The mean birth weight and gestational weeks at birth were 3284.84 ± 372.04 g and 38.94 ± 1.67 weeks, respectively. The proportion of cesarean-section (50.27%) in the obese group was significantly higher than those in the other BMI groups (20.78% to 37.97%). The mean birth weight increased over BMI, and it was 3314.41 ± 410.55 g in the obese group (Table 2).

**Table 2:** Infant characteristics and the prevalence of pre-pregnancy BMI.

Characteristics	BMI < 18.5 kg/m^2^ (n = 54,238)			18.5 kg/m^2^ ≤ BMI < 24.0 kg/m^2^ (n = 282,480)		24.0 kg/m ^2^ ≤ BMI < 28.0 kg/m ^2^(n = 51,251)			BMI ≥ 28.0 kg/m ^2^ (n = 10,399)			total		X^2^	*P*
	n	%	Prevalence	n	%	n	%	Prevalence	n	%	Prevalence	n	%		
			(%)					(%)			(%)				
**Childbirth**															
Vaginal birth	42,968	79.22	14.77	210,973	74.69	31,793	62.03	10.93	5172	49.73	1.78	290,906	73.02	7459.063	< 0.001
Cesarean-section	11,270	20.78	10.49	71,507	25.31	19,458	37.97	18.11	5227	50.27	4.86	107,462	26.98		
**Sex**															
Male	28,200	51.99	13.48	148,326	52.51	27,184	53.04	12.99	5506	52.95	2.63	209,216	52.52		
Female	25,985	47.91	13.77	133,855	47.39	24,009	46.85	12.72	4876	46.89	2.58	188,725	47.37	16.451	< 0.001
Unknown	53	0.1	12.41	299	0.11	58	0.11		17	0.16		427	0.11		
**Outcome**															
Survival	54,027	99.61	13.62	281,182	99.54	50,991	99.49	12.86	10,337	99.4	2.61	396,537	99.54		
Stillbirth	188	0.35	11.92	1119	0.4	218	0.43	13.82	52	0.5	3.3	1577	0.4	17.047	< 0.001
Died 7 days after birth	16	0.03	8.12	140	0.05	34	0.07	17.26	7	0.07	3.55	197	0.05		
Died between 7 and 42 days after birth	7	0.01	12.28	39	0.01	8	0.02	14.04	3	0.03	5.26	57	0.01		
Gestational weeks	38.96 ± 1.63			38.96 ± 1.66		38.89 ± 1.75			38.80 ± 1.81			38.94 ± 1.67		55.179	< 0.001
Birth weight	3262.61 ± 365.45			3284.84 ± 369.02		3304.88 ± 384.42			3314.41 ± 410.55			3284.84 ± 372.04		149.441	< 0.001

### Relationships between pre-pregnancy BMI and pregnancy/perinatal outcomes

The unadjusted and adjusted logistic regression results for the association of maternal pre-pregnancy BMI with pregnancy and perinatal outcomes are shown in Table 3. After adjusting for confounders, no significant associations were found between maternal pre-pregnancy BMI and birth defects, abortion, or stillbirth (all *P* > 0.05). Compared to the normal BMI group, the underweight group was associated with increased risk of LBW (AOR = 1.25, 95% CI 1.12–1.38) and SGA (AOR = 1.11, 95% CI 1.09–1.14), but protected against GHD (AOR = 0.85, 95% CI 0.75–0.95), GDM (AOR = 0.69, 95% CI 0.58–0.84), macrosomia (AOR = 0.67,95% CI 0.62–0.73),postterm pregnancy (AOR = 0.76, 95% CI 0.63–0.92), and cesarean-section (AOR = 0.81, 95% CI 0.76–0.84). Compared with the normal weight group, the overweight and obese groups were associated with increased risk of GHD (AOR = 1.28, 95% CI 1.16–1.43) (AOR = 2.47, 95% CI 2.24–2.74), GDM (AOR = 1.63,95% CI 1.35–1.93) (AOR = 3.02, 95% CI 2.12–3.79), preterm birth (AOR = 1.18, 95% CI 1.11–1.25) (AOR = 1.47, 95% CI 1.32–1.64), macrosomia (AOR = 1.51, 95% CI 1.42–1.60) (AOR = 2.11, 95% CI 1.90–2.35), LGA (AOR = 1.19, 95% CI 1.16–1.23) (AOR = 1.35,95% CI 1.27–1.42), postterm pregnancy (AOR = 1.39, 95% CI 1.19–1.63) (AOR = 1.66, 95% CI 1.25–2.21), and cesarean-section (AOR = 1.60, 95% CI 1.52–1.67) (AOR = 2.05, 95% CI 1.95–2.14), but protected against SGA (OR = 0.91, 95% CI 0.89–0.93) (OR = 0.90, 95% CI 0.86–0.94).

**Table 3:** Relationships between pre-pregnancy BMI and outcomes.

Outcomes	Unadjusted RR		Adjusted RR*	
	OR (95% CI)	*P*	AOR (95% CI)	*P*
**GHD**				
BMI < 18.5 kg/m^2^	0.84 (0.75–0.94)	0.003	0.85 (0.75–0.95)	0.005
18.5 kg/m^2^ ≤ BMI < 24.0 kg/m ^2^	1		1	
24.0 kg/m ^2^ ≤ BMI < 28.0 kg/m ^2^	1.26 (0.94–1.69)	0.122	1.28 (1.16–1.43)	0.004
BMI ≥ 28.0 kg/m ^2^	2.51 (2.22–2.89)	0.001	2.47 (2.24–2.74)	< 0.001
**GDM**				
BMI < 18.5 kg/m^2^	0.66 (0.55–0.79)	< 0.001	0.69 (0.58–0.84)	< 0.001
18.5 kg/m^2^ ≤ BMI < 24.0 kg/m ^2^	1			
24.0 kg/m ^2^ ≤ BMI < 28.0 kg/m ^2^	1.52 (1.27–1.83)	< 0.001	1.63 (1.35–1.93)	< 0.001
BMI ≥ 28.0 kg/m ^2^	3.06 (2.36–3.98)	0.002	3.02 (2.12–3.79)	< 0.001
**Preterm birth**				
BMI < 18.5 kg/m^2^	1.02 (0.96–1.08)	0.554	1.42 (1.26–1.60)	0.193
18.5 kg/m^2^ ≤ BMI < 24.0 kg/m ^2^	1		1	
24.0 kg/m ^2^ ≤ BMI < 28.0 kg/m ^2^	1.22 (1.15–1.29)	< 0.001	1.18 (1.11–1.25)	< 0.001
BMI ≥ 28.0 kg/m ^2^	1.54 (1.38–1.71)	< 0.001	1.47 (1.32–1.64)	< 0.001
**LBW**				
BMI < 18.5 kg/m^2^	1.20 (1.08–1.32)	< 0.001	1.25 (1.12–1.38)	0.024
18.5 kg/m^2^ ≤ BMI < 24.0 kg/m ^2^	1		1	
24.0 kg/m ^2^ ≤ BMI < 28.0 kg/m ^2^	0.89 (0.76–1.01)	0.192	0.90 (0.78–1.02)	0.201
BMI ≥ 28.0 kg/m ^2^	0.98 (0.86–1.12)	0.746	0.92 (0.80–1.05)	0.206
**Macrosomia**				
BMI < 18.5 kg/m^2^	0.67 (0.62–0.73)	< 0.001	0.67 (0.62–0.73)	< 0.001
18.5 kg/m^2^ ≤ BMI < 24.0 kg/m ^2^	1		1	
24.0 kg/m ^2^ ≤ BMI < 28.0 kg/m ^2^	1.52 (1.43–1.61)	< 0.001	1.51 (1.42–1.60)	< 0.001
BMI ≥ 28.0 kg/m ^2^	2.10 (1.89–2.34)	< 0.001	2.11 (1.90–2.35)	< 0.001
**SGA**				
BMI < 18.5 kg/m^2^	1.12 (1.09–1.4)	< 0.001	1.11 (1.09–1.14)	< 0.001
18.5 kg/m^2^ ≤ BMI < 24.0 kg/m ^2^	1		1	
24.0 kg/m ^2^ ≤ BMI < 28.0 kg/m ^2^	0.90 (0.86–0.95)	< 0.001	0.91 (0.89–0.93)	< 0.001
BMI ≥ 28.0 kg/m ^2^	0.91 (0.89–0.93)	< 0.001	0.90 (0.86–0.94)	< 0.001
**LGA**				
BMI < 18.5 kg/m^2^	0.87 (0.84–0.90)	< 0.001	0.87 (0.84–0.90)	< 0.001
18.5 kg/m^2^ ≤ BMI < 24.0 kg/m ^2^	1		1	
24.0 kg/m ^2^ ≤ BMI < 28.0 kg/m ^2^	1.20 (1.16–1.23)	< 0.001	1.19 (1.16–1.23)	< 0.001
BMI ≥ 28.0 kg/m ^2^	1.35 (1.28–1.43)	< 0.001	1.35 (1.27–1.42)	< 0.001
**Birth defects**				
BMI < 18.5 kg/m^2^	0.98 (0.88–1.09)	0.715	0.99 (0.89–1.36)	0.973
18.5 kg/m^2^ ≤ BMI < 24.0 kg/m ^2^	1		1	
24.0 kg/m ^2^ ≤ BMI < 28.0 kg/m ^2^	1.06 (0.94–1.18)	0.334	1.04 (0.93–1.17)	0.513
BMI ≥ 28.0 kg/m ^2^	1.09 (0.86–1.38)	0.49	1.05 (0.83–1.34)	0.666
**ABORTION**				
BMI < 18.5 kg/m^2^	0.96 (0.76–1.20)	0.717	0.99 (0.78–1.24)	0.949
18.5 kg/m^2^ ≤ BMI < 24.0 kg/m ^2^	1		1	
24.0 kg/m ^2^ ≤ BMI < 28.0 kg/m ^2^	1.11 (0.89–1.38)	0.363	1.02 (0.87–1.34)	0.514
BMI ≥ 28.0 kg/m ^2^	1.42 (1.95–2.13)	0.087	1.40 (0.93–2.10)	0.103
**Still birth**				
BMI < 18.5 kg/m^2^	0.76 (0.59–0.97)	0.028	0.82 (0.65–1.05)	0.119
18.5 kg/m^2^ ≤ BMI < 24.0 kg/m ^2^	1		1	
24.0 kg/m ^2^ ≤ BMI < 28.0 kg/m ^2^	1.13 (0.92–1.39)	0.025	1.07 (0.86–1.33)	0.536
BMI ≥ 28.0 kg/m ^2^	1.27 (0.85–1.92)	0.247	1.20 (0.79–1.81)	0.38
**Post-term pregnancy**				
BMI < 18.5 kg/m^2^	0.80 (0.67–0.96)	0.019	0.76 (0.63–0.92)	0.005
18.5 kg/m^2^ ≤ BMI < 24.0 kg/m ^2^	1		1	
24.0 kg/m ^2^ ≤ BMI < 28.0 kg/m ^2^	1.37 (1.17–1.59)	< 0.001	1.39 (1.19–1.63)	0.006
BMI ≥ 28.0 kg/m ^2^	1.61 (1.21–2.15)	0.001	1.66 (1.25–2.21)	< 0.001
**Caesarean-section**				
BMI < 18.5 kg/m^2^	0.83 (0.79–0.87)	< 0.001	0.81 (0.76–0.84)	< 0.001
18.5 kg/m^2^ ≤ BMI < 24.0 kg/m ^2^	1		1	
24.0 kg/m ^2^ ≤ BMI < 28.0 kg/m ^2^	1.70 (1.63–1.77)	< 0.001	1.60 (1.52–1.67)	< 0.001
BMI ≥ 28.0 kg/m ^2^	2.09 (2.00–2.19)	< 0.001	2.05 (1.95–2.14)	< 0.001

**Pre-pregnancy BMI was adjusted for maternal age, ethnic group, education, area of residence, occupation, smoking, passive smoking, alcohol drinking, and sex of children.

## Discussion

BMI is the most widely used measure to define obesity in terms of body weight and height. In addition, the fat mass index (FMI, kg of fat mass/ht^2^) and the fat-free mass index (FFMI, kg of fat-free mass/ht^2^) can also be used as indicators of nutritional status^12,13^. Since our study cohort from the FPHC surveillance system was measured by BMI only, we chose to use BMI in the present study. There are several methods for classification of BMI. For example, the Institute of Medicine (IOM) classified BMI ≥ 30.0 kg/m^2^ as obese in 2009^14^; the 1959 Metropolitan Standards recommended by Abrams and Parker classified BMI < 20 kg/m^2^ as underweight^15^. However, the figures of Chinese people are usually thinner especially in the south of China compared to those of people in many developing countries. A classification of BMI < 18.5 kg/m^2^ and ≥ 28.0 kg/m^2^ for underweight and obesity, respectively, was appropriate for our study.

In this retrospective study with a large cohort, we first investigated the prevalence of pre-pregnancy BMIs and the associations between pre-pregnancy BMI groups (especially the underweight group, BMI < 18.5 kg/m^2^) and pregnancy outcomes among women in Hunan Province, China. Our results showed that underweight women accounted for 13.62% of the cohort and that underweight occurred more often in young women, especially in individuals aged 20–24 years (17.98%), highly educated, institutional employed, and rural-based. The proportion of underweight women in Hunan Province was slightly higher than the average in China (13.62% versus 11%) and was lower than that in developing countries, such as India (21%)^16^, Thailand (18%)^17^, and Iran (16%)^18^, where undernutrition and infectious diseases are priorities^19^. Hunan is in southern China where rice is the staple food, which contains twice the amount of water and half of the energy as the same amount of steamed bread consumed in northern China^20^. Dietary restriction, exercise and attempting to attain a certain body type are the important reasons for underweight among young women in China^21^. The proportion of overweight/obese (15.48%) women in Hunan Province was consistent with the average in China, but is much lower than that in high-income countries, which was reported to be approximately 55.8% in the USA^22,23^. The results showed that women who were over 35 years old, low-educated, living in urban areas, and housewives were more likely to be overweight/obese. For older women, insulin resistance and higher ectopic fat accumulation may play a role^24^. For low-educated women or housewives, lack the knowledge regarding weight control and lack of a strong desire to lose weight were factors that may be contributing to overweight/obesity. Compared with rural women, urban women do less physical activity and are more likely to consume fast food^19^.

To date, there have been many studies about the relationship between BMI and pregnancy outcomes. However, most studies ignored the association between underweight and pregnancy outcomes. In our study, underweight mothers were found to be at higher risk of having LBW and SGA babies than normal-weight mothers. This finding was consistent with the results from previous studies with large Chinese cohorts^5,10,25^and systematic reviews^22^. Perhaps intrinsic maternal control of fetal size is correlated with maternal size in pregnancies^26^. Small uterus size and lower blood flow, found in short-statured women, impose direct physical limitations on the growth of the uterus, placenta and fetus. In addition, our study has added more results about the effects of underweight on pregnancy and perinatal outcomes in women. We found that underweight women were at lower risk of GHD, GDM, macrosomia, postterm pregnancy, and cesarean-section than normal-weight women. Perhaps for underweight women, it was less likely to develop impaired insulin secretion and glucose intolerance; they had lower mean arterial pressure and uterine artery pulsatility index^27^. Additionally, several studies showed a stepwise decrease in the prevalence of cesarean-section with decreasing BMI^4^.

After adjusting for confounding factors, this study showed that overweight and obesity increased the risk of GHD, GDM, preterm birth, macrosomia, LGA, postterm pregnancy,and cesarean-section. These findings are consistent with those in the previous studies. Regarding GHD, obese women have been shown to have an increased blood volume and cardiac output and increased blood pressure during pregnancy^28^. Regarding GDM, macrosomia and LGA, the mechanism may be related to insulin resistance, which is often present in women with obesity or excessive weight gain during pregnancy. Insulin resistance causes metabolic disorders, resulting in the increased availability of nutrients to the fetus, which receives large amounts of glucose through the placenta, leading to hyper-insulinemia and fetal growth acceleration^10^. The larger size of the fetus also increases the difficulty of natural labor. In addition, the study by Wu et al. showed lower odds of attempting assisted vaginal birth in obese women than in normal-weight women^4^.

Few studies have investigated the associations of maternal pre-pregnancy BMI with the pregnancy outcomes including birth defects, stillbirth and abortion. The statistical findings of this study suggest that the associations of pre-pregnancy BMI with these outcomes were non-significant. However, there are inherent limitations to the present study. A proportion of birth defects, stillbirth, and abortion occurred in early pregnancy before 12 gestational weeks might fail to report to the surveillance system, as these women did not need inpatient service in the local maternal and child health care hospital. As a result, the sample sizes of these events were insufficient to determine the difference between the distinct BMI groups. Therefore, it is worthwhile to further investigate the associations between pre-pregnancy BMI and the adverse outcomes including birth defects, stillbirth and abortion.

## Data Availability

The data analysed during the current study are available from the corresponding author on reasonable request. The data are not publicly available due to privacy.
